# On the facilitative effects of face motion on face recognition and its development

**DOI:** 10.3389/fpsyg.2014.00633

**Published:** 2014-06-24

**Authors:** Naiqi G. Xiao, Steve Perrotta, Paul C. Quinn, Zhe Wang, Yu-Hao P. Sun, Kang Lee

**Affiliations:** ^1^Department of Psychology, Zhejiang Sci-Tech UniversityHangzhou, China; ^2^Applied Psychology and Human Development, University of TorontoToronto, ON, Canada; ^3^Department of Psychology, University of DelawareNewark, DE, USA

**Keywords:** facial movement, elastic facial movement, rigid facial movement, face processing, holistic face processing, part-based face processing, eye movements, development of face processing

## Abstract

For the past century, researchers have extensively studied human face processing and its development. These studies have advanced our understanding of not only face processing, but also visual processing in general. However, most of what we know about face processing was investigated using static face images as stimuli. Therefore, an important question arises: to what extent does our understanding of static face processing generalize to face processing in real-life contexts in which faces are mostly moving? The present article addresses this question by examining recent studies on moving face processing to uncover the influence of facial movements on face processing and its development. First, we describe evidence on the facilitative effects of facial movements on face recognition and two related theoretical hypotheses: the supplementary information hypothesis and the representation enhancement hypothesis. We then highlight several recent studies suggesting that facial movements optimize face processing by activating specific face processing strategies that accommodate to task requirements. Lastly, we review the influence of facial movements on the development of face processing in the first year of life. We focus on infants' sensitivity to facial movements and explore the facilitative effects of facial movements on infants' face recognition performance. We conclude by outlining several future directions to investigate moving face processing and emphasize the importance of including dynamic aspects of facial information to further understand face processing in real-life contexts.

Human faces are perhaps the most important class of visual stimuli in our environment. Every day we encounter faces. These faces change viewpoints, present different expressions, and convey crucial linguistic information. The human face also provides an observer with characteristic identifying information, such as age, sex, race, and identity itself. Through our experience with faces in social interactions, we may develop sophisticated abilities to process faces in a highly efficient way.

For decades, researchers have extensively studied human face processing and its development. These investigations have advanced our understanding of not only face processing, but also visual processing in general (e.g., Calder et al., 2011). It is worth noting that most of what we know about face processing has been derived from studies that have exclusively used static face images; however, the faces we encounter in the real world are constantly moving. Therefore, researchers have raised a question about to what extent our understanding of face processing using static face stimuli can be generalized to the moving faces that we see in our daily lives. Two types of facial movement exist: rigid movement and elastic movement (O'Toole et al., 2002). Rigid facial movement refers to transient changes in face orientation while facial structure remains unchanged (e.g., head turning and nodding). Elastic facial movement refers to the structural transformation of the facial skeletal musculature (e.g., expressions, eye gaze changes; Figures 1, 2). In many social situations, human faces present both types of facial movement concurrently (Bruce and Valentine, 1988; O'Toole et al., 2002).

**Figure 1:**
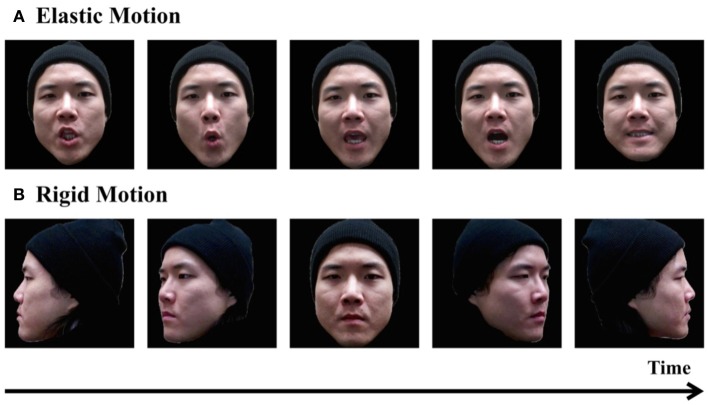
**Demonstrations of elastic facial motion (A) and rigid facial motion (B)**.

**Figure 2:**
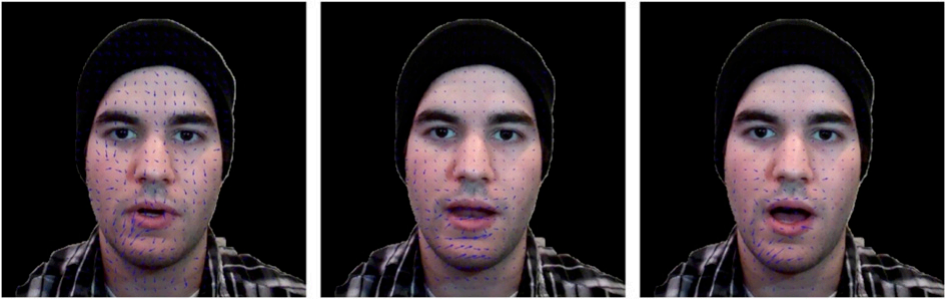
**Optical flow patterns for elastic facial movements at different time points**. The blue arrows indicate direction and magnitude of facial movements. The motion direction and magnitude vary in different face areas and time points.

Recently, researchers have begun to examine the role of facial motion on face processing to obtain a more comprehensive account of face perception (Knight and Johnston, 1997; Pike et al., 1997; Hill and Johnston, 2001; Wallis and Bülthoff, 2001; O'Toole et al., 2002; Thornton and Kourtzi, 2002; Knappmeyer et al., 2003; Lander and Bruce, 2003; Pilz et al., 2006; Otsuka et al., 2009; Ichikawa et al., 2011; Xiao et al., 2012, 2013b). Most of these studies have observed that learning a face in motion leads to better recognition than learning static faces, a phenomenon called the facial movement beneficial effect (O'Toole et al., 2002). This effect suggests that the mechanisms underlying face motion processing may differ from those underlying static face processing. In the current paper, we will review investigations on the role of face movements in face recognition and its development. We will first introduce literature on the facilitative effect that facial movements have on recognition, and discuss two related theoretical hypotheses. Then, we will discuss recent studies indicating that facial movement optimizes face processing. Lastly, we will consider findings regarding facial movement's contributions to the development of face processing in the first year of life.

In the dynamic face processing literature, facial movements have been classified into two major categories: elastic and rigid facial movement. Most studies have focused on faces depicting elastic facial movement, and very few have indicated a fundamental difference between elastic and rigid facial movements in terms of their influences on face processing (see Lander and Bruce, 2003). Thus, the present review focuses on the commonalities of the effects of elastic and rigid facial movements without discussing the differences between them.

## Facial motion improves face recognition

The facilitative effects of facial motion were first reported by Knight and Johnston (1997). In this study, participants viewed either a video or a static image of a famous person presented in negative black–white contrast. Recognition performance was better in the video condition than in the static image condition. Since the publication of this initial finding, other researchers have consistently observed beneficial effects for facial movement in recognizing famous faces, familiar faces, and unfamiliar faces (Pike et al., 1997; Lander et al., 1999, 2004; Lander and Bruce, 2000, 2003, 2004; Bruce et al., 2001; Thornton and Kourtzi, 2002; Pilz et al., 2006; Roark et al., 2006; Lander and Davies, 2007; O'Toole et al., 2011; Arnold and Siéroff, 2012). Based on this body of work, researchers have proposed two mechanisms regarding facial motion's enhancement of recognition: one suggesting that facial movement provides idiosyncratic facial information in addition to static facial information (the supplementary information hypothesis), and the other suggesting that facial movements assist in producing a more robust and flexible three-dimensional face representation in learning new faces, thus improving face recognition (the representation enhancement hypothesis: O'Toole et al., 2002; O'Toole and Roark, 2010). In the following sections, we will discuss these two hypotheses and their supporting evidence.

### Facial movements provide additional facial information

The idea that motion contains identity information has been proposed in early studies using point-light stimuli (Johansson, 1973). Subsequent investigations have consistently found that facial movements contain rich information, including facial expression, age, sex, and identity (e.g., Bassili, 1978; Berry, 1990; Rosenblum et al., 2002). For example, Hill and Johnston (2001) found that observers could accurately judge gender and identity from facial movement patterns alone. Similarly, Knappmeyer et al. (2003) reported that observers relied on the resemblance of elastic facial movement patterns to infer identity and kinship between two moving faces. These studies suggest that we are sensitive to facial movements, which contain identifying information. Among the various aspects of facial information embedded in facial movements, some researchers have argued that idiosyncratic dynamic facial information can be used to assist face recognition (the supplementary hypothesis, O'Toole et al., 2002). When a moving face is presented, observers can recognize this face not only based on static facial information, such as facial features and spatial configuration, but also based on idiosyncratic motion information, such as the way an individual typically smiles.

**Figure 3:**
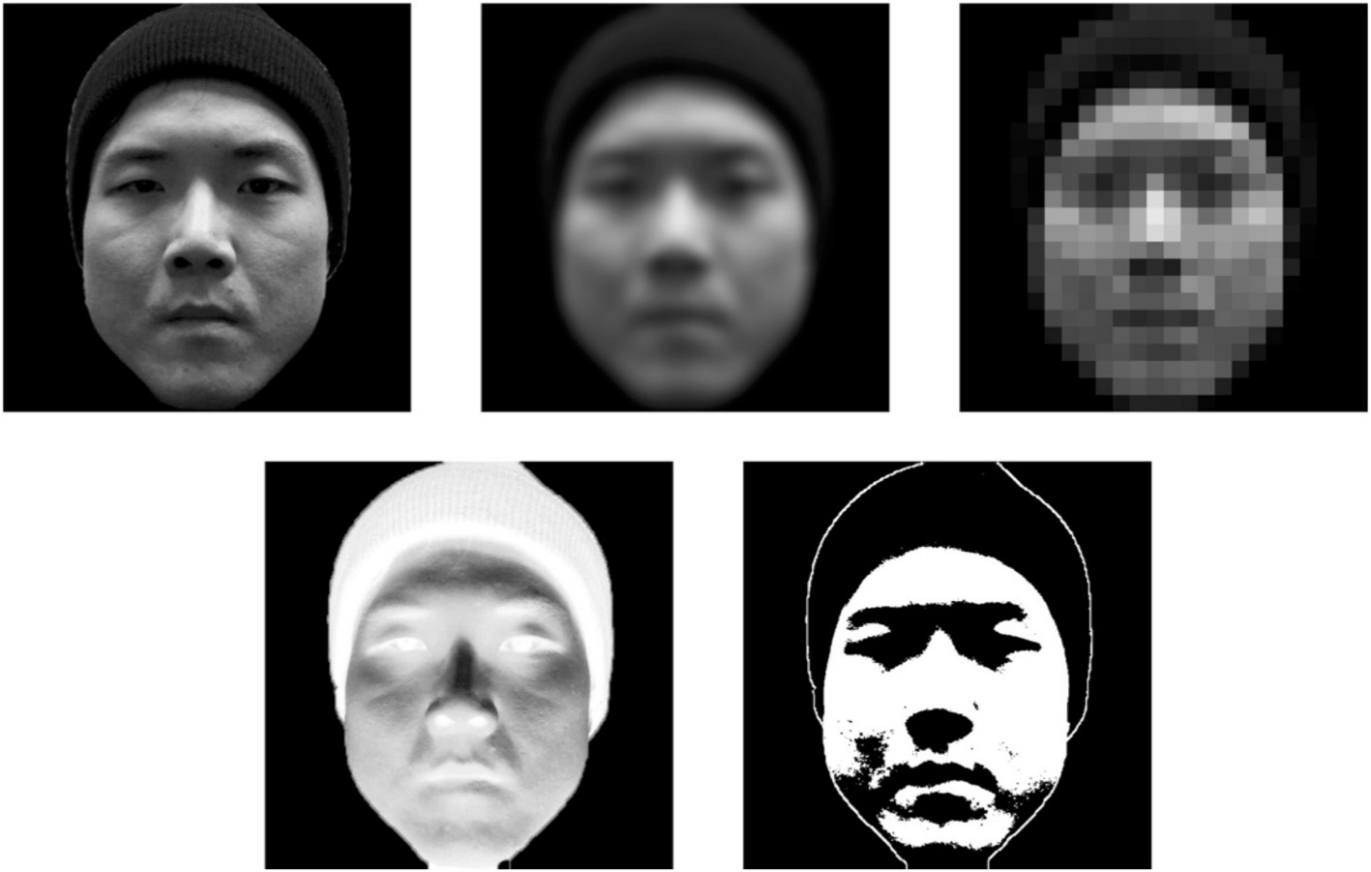
**The demonstration of face images under different non-optimal viewing conditions**. The normal image (**left panel** of 1st row), the blurred image (**middle panel** of 1st row), the pixelation image (**right panel** of 1st row), negation image (**left panel** of 2nd row), and the thresholding image (**right panel** of 2nd row).

Research has revealed that observers use facial movement information only when the static facial information is not informative. In a previously mentioned study, Knight and Johnston (1997) compared recognition performance between videos and static images. They found a recognition enhancement for facial movement only when videos were presented in negative black–white contrast, but not in standard contrast. These results suggest that facial movements contribute to face recognition under non-optimal viewing conditions, but not under normal viewing conditions. Knight and Johnston (1997) suggest that static facial information becomes difficult to access under non-optimal conditions, implying that this information is not sufficient for recognition. Therefore, facial movements supply the visual system with additional facial information to assist in recognition. Consistent with this observation, researchers have reported the facilitation effect of facial movements under other non-optimal viewing conditions, such as image blurring, pixelation, negation, and thresholding (Knight and Johnston, 1997; Burton et al., 1999; Lander et al., 2001, see review in Roark et al., 2003; Figure 3). Taken together, these studies indicate that the information embedded in facial motion is useful for facilitating recognition only when there is interference with static facial information processing. In other words, idiosyncratic dynamic facial information serves a supplementary role to static facial information in face recognition.

Further examination of the beneficial effect of face motion has revealed that enhanced recognition relies on the naturalness of facial movements. For example, Lander and Bruce (2004) reported that famous faces presented in slow motion led to poorer recognition than those presented in normal speed. Lander and Bruce (2000) also observed higher face recognition performance when videos were played at their natural speed than when they were sped up, even when the sped-up videos were played twice so that an equal amount of information was present in both the natural and altered videos. Lander et al. (2006) further examined the effect of naturalness by morphing smiles of faces that were personally familiar to their participants. They found that recognition was better when faces were shown smiling than when displaying a neutral expression that was morphed into a smile. Furthermore, increasing the speed of the smile impaired recognition performance for the natural smile only (Lander et al., 2006). In line with these studies, Hill and Johnston (2001) demonstrated that reversing the facial movement video significantly impaired face gender judgments. This behavioral evidence was further supported in a recent neuroimaging study. Schultz et al. (2013) observed that activation in the superior temporal sulcus (STS) region was modulated by facial movement presentation fluidity; the activation in the STS became stronger as facial movements appeared more fluid. Taken together, these findings indicate that naturalness of facial motion is a critical factor that contributes to the enhanced recognition effect. These studies further suggest that the facilitation effect is derived from representations of facial movements. When the presentation of facial movements deviates from the way those movements are normally perceived (e.g., by altering motion speed), recognition becomes impaired.

The supplementary information hypothesis suggests that facial movement and static information are processed independently, and therefore may be processed in different brain areas. As posited in the distributed face processing model, static face information is processed mainly in the ventral pathway, which includes the fusiform face area (FFA) and the occipital face area (OFA). Facial movement information is processed in the dorsal pathway, which includes the STS region (Haxby et al., 2000). The supplementary hypothesis assumes that deficits in static facial information processing are not linked to impairments in facial movement processing.

Prosopagnosia is a disorder that causes face recognition impairment, while other aspects of visual and cognitive processing, such as object discrimination and intellectual functioning, remain intact (for a review, see Bate, 2013). Recent studies of prosopagnosic patients support the supplementary hypothesis by suggesting that the dynamic aspect of facial information is stored independently of static facial information. Steede et al. (2007) examined CS, a man born with prosopagnosia. Through a series of tests, CS was found to discriminate between faces when presented in motion. In addition, CS was required to match names to moving faces and discriminate faces based on their facial movement patterns. Most importantly, CS exhibited recognition performance almost at the same level as a control group when faces were presented in motion. These findings were further supplemented by a recent prosopagnosia study in which Longmore and Tree (2013) tested four congenital prosopagnosia patients and found that they were able to use facial movements as a cue to recognize faces, in spite of their impairment in static face recognition. These studies support the supplementary hypothesis, in that there exists a dissociation between static and dynamic aspects of facial information processing.

It should be mentioned that the two prosopagnosia studies above only included congenitally prosopagnosic patients, rather than acquired prosopagnosic patients. As noted in O'Toole and Roark (2010), the observed dissociation between static and dynamic facial information processing reflects the possibility that the ability to process facial movements has been developed to compensate for impaired static face processing. One study found that an acquired prosopagnosic patient exhibited deficits in abilities required to recognize faces from both static and dynamic facial information (Lander et al., 2004). More studies on acquired prosopagnosia patients are needed to further understand the role of facial movements in face recognition.

In summary, when recognizing familiar moving faces, facial movements can provide additional identifying information to assist face recognition (a dynamic facial identity). The dynamic identity serves a supplemental, but independent, role relative to static facial information for face recognition. The effect of dynamic facial identity is revealed mainly when static facial information becomes uninformative and when faces are familiar to observers. The naturalness of facial movements, which includes both temporal and spatial characteristics, is crucial to its facilitation effect in recognition. The hypothesis of the dynamic facial identity (i.e., the supplemental information hypothesis) has received supportive evidence from studies in both the normal population and in patients with congenital prosopagnosia. It should be noted that the studies we reviewed above examined the role of facial movement in recognizing familiar faces. They were unable to reveal whether facial movement facilitates learning a new face. In the next section, we will review the representation enhancement hypothesis to account for how facial movement facilitates face learning.

### Facial movements enhance face representation

Researchers have observed that facial movements also improve learning new/unfamiliar faces. Studies have shown that learning new faces in motion leads to better recognition performance than learning the face from a static image. This facial motion facilitation effect in learning new faces suggests that facial movements assist in forming a robust face representation, which in turn leads to enhanced face recognition performance (the representation enhancement hypothesis, O'Toole et al., 2002; O'Toole and Roark, 2010).

Support for the representation enhancement hypothesis is shown when faces are recognized more accurately when they are learned in motion rather than from single or multiple static face images (O'Toole and Roark, 2010). For example, Pike et al. (1997) presented participants with rigidly moving face videos (i.e., head rotation), or multiple static face pictures extracted from the videos. Participants were then shown an image of the same face, or a novel face. Recognition performance was significantly better when participants learned the face from rigid motion than from multiple images. Lander and Bruce (2003) used a similar paradigm to reveal a recognition facilitation effect from elastic facial movements (i.e., speaking and smiling). The authors suggested that this motion facilitation effect may be due to the continuity of movements, rather than to the fact that moving videos contain more static facial information (i.e., more image frames).

In addition to the facilitation effect of motion observed in studies using an old/new paradigm, which reflects an effect of long-term memory, researchers have also reported that facial movements facilitate face recognition in a relatively short period of time. For example, Thornton and Kourtzi (2002) used a face-matching paradigm in which participants were instructed to remember faces presented in elastic motion or as a static image, and were then tested with a static face image. Participants responded faster in the test when the previous face was presented in motion. The results suggest that, even within a short amount of time, learning faces in motion can improve recognition of static faces. In other words, facial movements enhanced face representation when observers first learn the face in motion. In the following studies, Pilz et al. (2006) and Pilz et al. (2009) demonstrated similar facilitation effects using visual search and face matching tasks. Moreover, Pilz et al. (2006) observed that learning moving faces led to advanced recognition performance even when the test face was presented in a different viewpoint from that of the learned moving face. This outcome further suggests that the facial representation as enhanced by motion may be a three-dimensional representation, which is robust and flexible to viewpoint changes.

To summarize, although a few early investigations failed to observe a facilitation effect from learning moving faces (e.g., Christie and Bruce, 1998), most studies have consistently demonstrated this facilitation in recognizing static faces, which do not contain any motion information. This facilitated recognition suggests a role for facial movement in forming a more robust face representation. This effect may be due to the fact that moving faces convey stronger social signals (e.g., speaking and expression), which may attract more attention than static faces, thereby forming a more robust face representation (Lander and Bruce, 2003). It should be noted that learning a moving face might also involve forming a dynamic idiosyncratic facial representation. This dynamic facial representation might be not as important as an enhanced face representation in improving face learning (e.g., Butcher et al., 2011). As the facilitative effect of face movement derives mostly from an enhancement of a three-dimensional face representation, it does not require degrading static facial information.

Both the supplementary information hypothesis and representation enhancement hypothesis have illustrated that facial motion plays critical roles in learning new/unfamiliar faces and recognizing familiar faces. Facial movement provides rich information about a new face, which allows one to form an enhanced face representation. Once a face becomes familiar, its idiosyncratic facial movement pattern can serve as a cue for identity to facilitate recognition, especially when facial static information becomes uninformative under certain viewing conditions. However, it should be noted that most of the existing studies examined the enhanced face representation in learning new moving faces, and the idiosyncratic facial movements in recognizing familiar faces. This does not necessarily suggest that the enhanced face representation by facial movements would not facilitate familiar face recognition. It is also unclear whether idiosyncratic facial movement affects face learning. In addition, it should be noted that most studies have focused on the effects of facial movement on face representation. It is still unclear as to what the underlying mechanisms are that allow an enhanced face representation to be formed by facial movements. To further probe this important issue, we will review recent studies that focus on facial movement's influence on facial information encoding.

## Facial movements optimize face processing

Facial movements have been found to improve facial representations, thereby enhancing recognition performance. It is worthwhile to investigate whether or not this facilitation effect demonstrates that moving faces are processed in a more appropriate way than static faces. In this section, we will attempt to answer this question by reviewing recent studies on the influence of facial movements on face processing. Specifically, these studies attempted to determine whether facial motion would promote a more appropriate face processing mode than static images.

In the real world, we are constantly interacting with moving faces. These face-to-face interactions commonly include different kinds of tasks, such as recognizing a face, understanding facial expressions, and judging gender. Moreover, as face-to-face interaction progresses, the task may change. Thus, the changing demands of face-to-face interactions in real life situations require observers to voluntarily shift face processing strategies accordingly. However, in previous investigations using static face images, an inflexible, rather than flexible, face processing strategy has been observed (e.g., Young et al., 1987; Schweinberger and Soukup, 1998; Ganel et al., 2005). Observers are unable to adjust their face processing strategy to cope with the presence of irrelevant facial information, which can interfere with face recognition, reflecting rigidity in face processing (e.g., Schweinberger and Soukup, 1998; Ganel et al., 2005; Richler et al., 2008). For example, laboratory studies have shown that when processing face identity, observers are unable to avoid irrelevant emotional information (e.g., Ganel et al., 2005). This contrast between face processing in real-life situations and that reported in the literature leads us to ask whether the lack of flexibility in static face processing is related to the absence of facial movements. The studies to be reviewed in this section will shed some light on this question.

Previous face processing research suggests that there are two types of processing: holistic/configural face processing and part-based face processing. Holistic face processing consists of a tendency to integrate facial information within the whole face region as a gestalt. Part-based face processing demonstrates the tendency to process facial information individually, in a feature-by-feature manner. Studies have shown that normally-developed individuals process faces and face-like stimuli holistically (Young et al., 1987; Tanaka and Farah, 1993; Moscovitch et al., 1997; Tanaka et al., 1998; Maurer et al., 2002; Le Grand et al., 2004; Michel et al., 2006; Schwarzer et al., 2007; Richler et al., 2008, 2011a; Wang et al., 2012; Wong et al., 2012). Holistic face processing is primary for upright human face stimuli, but its use is greatly reduced for processing objects, inverted faces, or upright faces from another ethnic background (Michel et al., 2006; McKone et al., 2007, 2013; McKone, 2008). This specificity of holistic face processing reflects the visual system's adaptation to its unique visual environment. The consequence of this adaptation can be supported by recent findings suggesting that an individual's tendency to process faces holistically moderately predicts their face recognition abilities (Richler et al., 2011a; DeGutis et al., 2012; Wang et al., 2012). Neuropsychological studies also offer support for a holistic dominance hypothesis by demonstrating that patients with disorders related to face processing, such as prosopagnosia and autism spectrum disorder (ASD), exhibit weaker holistic face processing (Avidan et al., 2011; Weigelt et al., 2011). In summary, holistic face processing has been regarded as a hallmark of mature face processing in the literature.

**Figure 4:**
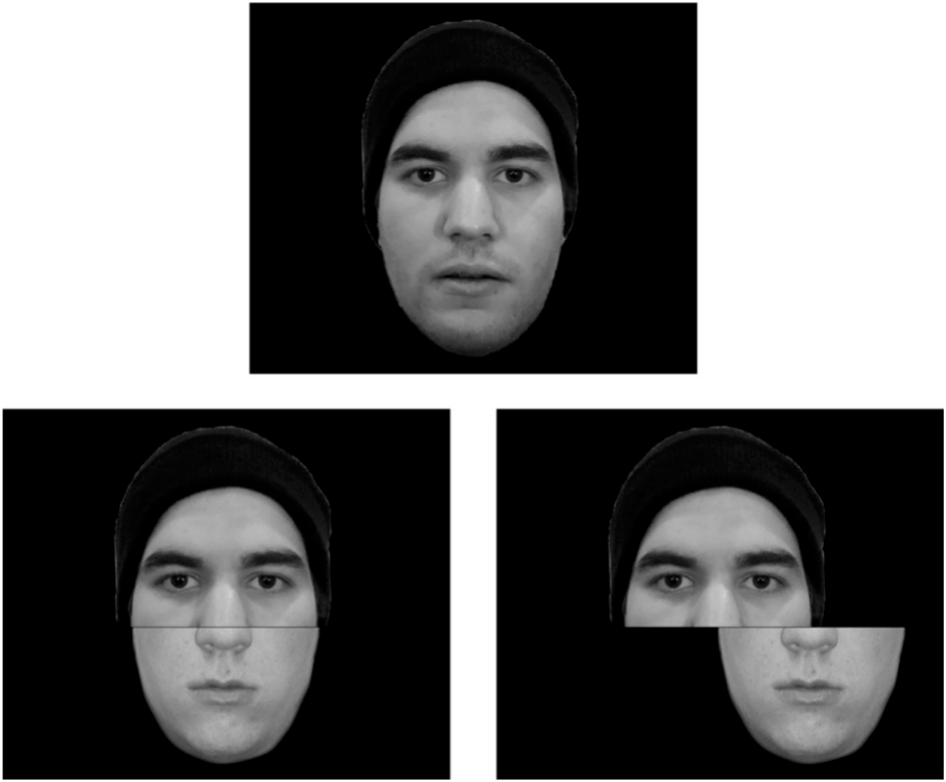
**Examples of original faces (upper), aligned (lower left panel), and misaligned (lower right panel) composite faces**. Observers find it difficult to judge whether the upper face of the original and aligned composite faces are the same person. By contrast, this judgment is relatively easy when the upper and lower face halves are misaligned.

In spite of the fact that holistic processing gives rise to sophisticated face processing, it can sometimes interfere with face processing. This interference is most obvious in face-part information processing, as demonstrated in the classical composite face effect (Young et al., 1987; Richler et al., 2011b, 2013). In the composite face effect, the recognition of a certain face part (e.g., the upper face half) is affected by the presence of another irrelevant face part (e.g., the lower face half). This phenomenon has usually been examined by using a composite face image, in which face parts from two separate faces (e.g., the upper half of face A and lower half of face B) comprise one whole face image. The most important manipulation is the alignment of these two face parts. For the aligned condition, the two face parts are aligned so as to be perceived as a whole face. For the misaligned condition, an offset exists between the two face parts so as to eliminate perception of a whole face (Figure 4). Participants' recognition performance for either face half is worse when the two face parts are aligned than when they are misaligned, which is the composite face effect. This effect suggests the dominance of involuntary holistic face processing. Even though participants were explicitly asked to ignore the irrelevant face part, the composite effect remained robust. This result also signifies the rigidity of face processing, in which observers are unable to ignore the irrelevant facial information. In this composite face task, an optimal strategy would be to utilize a part-based processing method, in which one focuses on the relevant face part information while disregarding the irrelevant face part information. However, due to the rigidity of holistic face processing, observers were unable to switch to a feature-based processing mode that could have led to optimal performance.

While studies consistently demonstrate the composite face effect using static face pictures as stimuli, recent studies from our lab indicate that this effect becomes weaker or even disappears when stimuli are comprised of moving faces. For example, Xiao et al. (2013b) used the composite face effect paradigm to examine whether elastic facial movement affects holistic face processing. In each trial, participants first learned a frontal-view face, followed by the presentation of a static composite face as a test stimulus. The composite face was comprised of the upper part of one face and the lower part of another. Participants were advised to ignore the lower face half, which was a different person from the learning face, acting as the irrelevant face information. They were asked to judge whether the upper part of the composite face belonged to the just learned face. The critical difference lay in the type of face learned; in half of the trials, participants learned the face with elastic facial movement, and in the other half, they learned the face via multiple static pictures. The moving face stimuli depicted blinking and chewing facial movements, whereas the static face pictures were frames extracted from the dynamic face videos.

**Figure 5:**
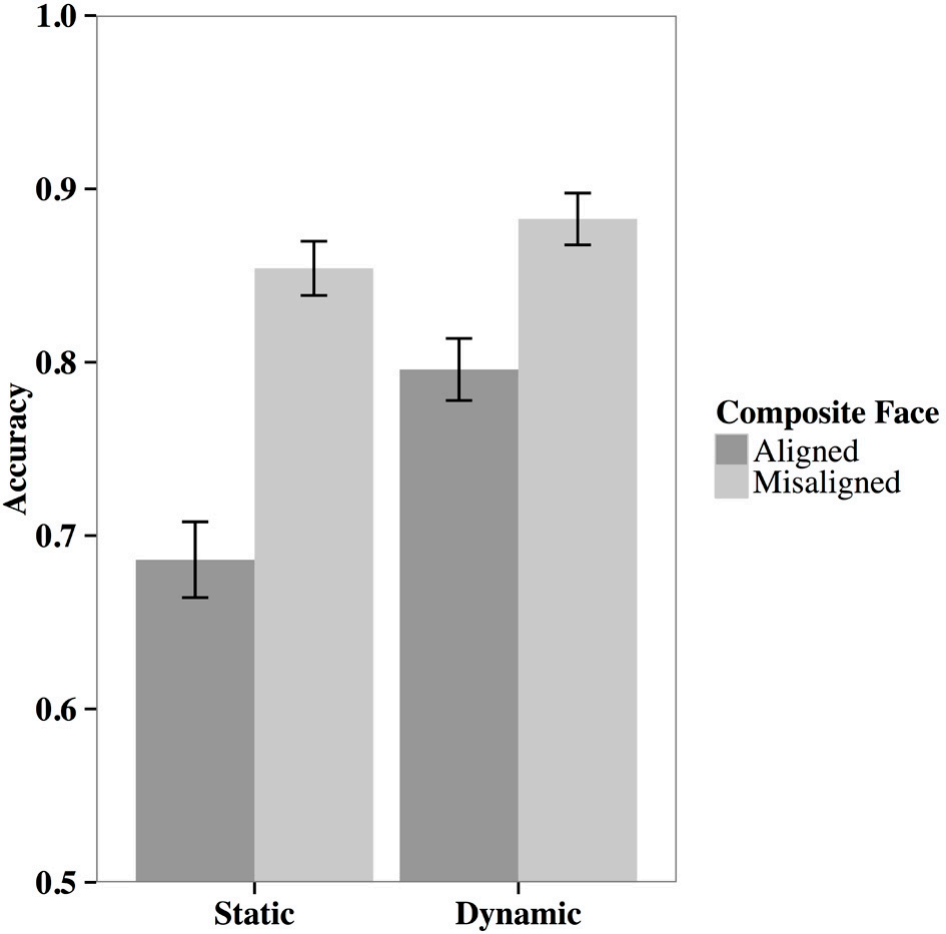
**The results from Xiao et al. (2013b) indicate that elastic facial movement led to smaller composite effects than static faces**. The findings suggest that elastic facial movement may result in greater part-based face processing.

The results of Xiao et al. (2013b) indicated that moving faces led to better recognition overall. More importantly, moving faces resulted in a significantly smaller composite effect than static face pictures (Figure 5). This finding suggests that participants' face part recognition was less affected by the irrelevant face part when they learned moving faces as opposed to static ones. In other words, when learning elastic moving faces, participants used more part-based processing, rather than holistic processing, to achieve better recognition performance, thereby adapting to the task requirement. Further analyses supported this argument by demonstrating that the advanced part-based face processing elicited by facial movements predicted the facial movement facilitation effect in face recognition. In an additional experiment, the results replicated when participants were asked to recognize the lower face part rather than the upper part. This outcome suggests that the facilitation of part-based processing by facial movement was not related to the specific characteristics of the facial movements, given that the lower face half movements were more salient than those in the upper half due to chewing. Instead, these results taken together indicate that facial movement modifies the way that facial information is processed, shifting face processing from holistic to part-based, thereby accommodating to the requirement that one should ignore the irrelevant face half.

The finding that elastic facial movement could facilitate part-based face processing has been further supported in studies of rigid facial movement. Xiao et al. (2012) compared the size of the composite effect for learning rigidly moving faces and static face images. The moving and static stimuli were comprised of identical face pictures from eight viewpoints. For the moving face stimuli, these face pictures were presented sequentially according to viewpoint angle, which could be perceived as a coherent face rotation movement. By contrast, in the static condition, these pictures were either displayed randomly or sequentially with intervals in between pictures, from which motion could not be perceived. Results indicated that the static face pictures led to a significant composite face effect, while no effect was observed in the dynamic condition. The findings suggest that the facilitative effect of facial movements in promoting part-based face processing in the composite face task was not limited to elastic moving faces; it can be generalized to rigid moving faces. These findings further indicate that this facilitation in face processing may be a general effect for various types of facial movements. In an even more recent study, the facial movement's part-based processing facilitation effect was observed in 8- and 12-year-olds (Xiao et al., 2013c), suggesting that the ability to use facial movements for face processing is already present in middle childhood.

To further understand the underlying mechanisms of the facilitative effect of facial movement, researchers recently used a high-frequency eye tracker to investigate whether the facilitation effect could be revealed in participants' eye movement patterns. Using a design identical to Xiao et al. (2013b), elastic moving faces were found to lead to visual fixations of longer duration than static faces (Xiao et al., 2014a). This result suggests that participants encode local facial information to a greater extent in moving faces than static ones. More importantly, a correlation was revealed between the part-based processing facilitation effect and observers' eye movement patterns. Each participant's upper face looking time advantage while learning moving relative to static faces positively predicted the part-based face processing increase engendered by facial movements. This association was only observed in the aligned but not the misaligned condition, indicating that fixating on the moving upper face half was specific to reducing the interference from the aligned lower face half. These findings indicate that facial movement enhances part-based face processing by influencing eye movement patterns, which further supports the proposal that facial movement facilitates facial information encoding to accommodate to task requirements. Taken together, these moving face studies challenge the rigidity of holistic face processing found in previous static face processing studies. It appears that facial movement tailors observers' face processing strategy to the task requirements, so as to reach better performance.

Besides holistic face processing, rigidity in face processing has also been revealed in other paradigms that used static faces. Previous studies have shown that facial identity and facial emotion information are often integrated during processing. For example, by using static face stimuli, Schweinberger and Soukup (1998) observed that facial emotion recognition was easily interfered with by facial identity information. A reverse form of interference in facial identity processing from emotion processing was also reported in studies using static familiar faces (Ganel et al., 2005). These studies suggest that it is difficult to selectively attend to either facial identity or emotional information while ignoring the other.

Rigidity between facial identity and emotion information processing has not, however, been observed in a recent study using moving faces as stimuli. Stoesz and Jakobson (2013) used the Garner classification task to measure the interference between facial identity and facial emotion processing. For each trial, one face was presented in the center of the screen, which could vary in two dimensions: identity (Ann or Jane) and emotion (anger or surprise). In the identity judgment task, participants were instructed to identify the face, while ignoring its facial expression. Participants first finished a baseline block to measure their response accuracy, in which facial expression was kept constant. An orthogonal block followed the baseline block, in which facial emotion would vary randomly. The interference score from facial emotion to facial identity processing was calculated by subtracting the accuracy in the orthogonal block from that in the baseline block. The interference score for identity processing was measured in the same way, in which the task was to judge facial emotional content while ignoring identity information. For half the trials, faces were presented in motion, while for the other half, they were presented as static pictures.

Consistent with previous findings, Stoesz and Jakobson (2013) reported that static faces showed significant interference from task-irrelevant facial information. Participants recognized face identity better when facial emotion was kept constant than when it varied. They also performed better in facial expression judgment when facial identity was unchanged, rather than when it was modified. However, participants' identity and expression judgments were unaffected by the irrelevant facial information's variation when faces were moving. Moreover, a recent study using similar methods replicated this finding that dynamic facial information reduced the interference between facial identity and emotion processing (Rigby et al., 2013). They further observed the facial movement effect when faces were upright, but not when they were inverted. This result suggests that facial movement might promote the switch from using a global processing strategy to a local processing strategy. These findings indicate that static facial identity information and emotional information may interfere with one another. The introduction of facial movement appears to promote separation in processing facial identity and emotion information, as is evident by the robust reduction in mutual interference.

Overall, to our knowledge, most of the current direct evidence regarding the effect of facial movement on face processing indicates a promotion of part-based or local oriented processing. This leaves us with a question of whether the effect of facial movement on face processing is purely a facilitation of part-based processing or a promotion of flexibility in face processing. One recent study has shown that facial movements affect gender judgments by producing a larger face inversion effect (Thornton et al., 2011), which reflects greater holistic processing (Yin, 1969; Gallay et al., 2006; Crookes and Hayward, 2012; Laguesse et al., 2012; Xu and Tanaka, 2013). In addition, another study reported that facial movement led to better emotional judgment than static faces, although the size of holistic face processing was comparable between the moving and static face conditions (Chiller-Glaus et al., 2011). These studies indicate that facial movement may facilitate or not affect holistic face processing under specific task requirements. Further, they suggest that motion exerts this effect by increasing the flexibility of face processing.

In addition to the flexibility reflected in holistic vs. part-based face processing, one might need flexibility in processing moving faces from different ethnic backgrounds. Previous studies have consistently shown that recognizing other race faces is more difficult than recognizing own race faces, a phenomenon known as the other race effect (ORE). Recent studies suggest that the ORE might be related to face scanning strategy; observers may be unable to allocate their visual fixation toward diagnostic information in other race faces (e.g., Caldara et al., 2010; Fu et al., 2012; Xiao et al., 2014b). To our knowledge very few studies have examined the ORE with moving faces. It is possible that facial movement might enhance the diagnostic information in other race faces, allowing for more efficient processing than in static faces.

To conclude, the studies discussed in this section have shown that facial movement may lead to face processing that is distinctive from that observed in prior studies using static face images as stimuli. The optimal face processing strategy observed for moving faces may reflect an optimization consequence of facial movements, which engenders adaptation to specific task requirements, therefore leading to better performance than that with static faces. Although the reviewed studies do not provide an overarching explanation for the role of facial movements in promoting face processing flexibility, one possible interpretation is that facial movements amplify the accessibility of different facial information. The increased accessibility may allow the visual system to select task-relevant facial information more readily. This argument is supported by a recent study showing that increasing the saliency of facial feature information alters face processing to utilize more facial featural information (Goffaux, 2012). It is necessary to examine different types of facial movements with various face-related tasks to further understand the role of facial movements in face processing optimization.

**Figure 6:**
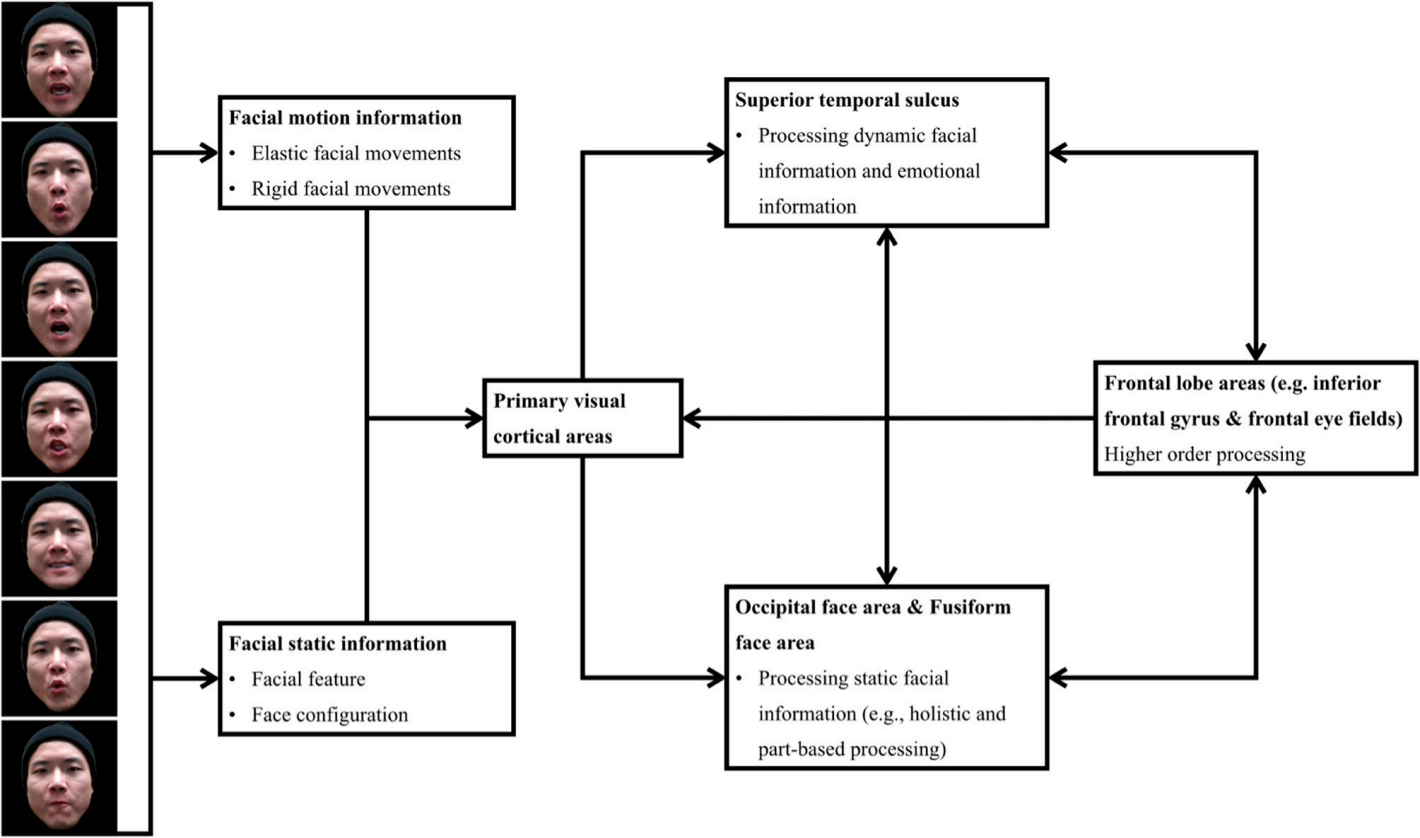
**A model of the neural system for processing moving face**.

Most of the existing findings on face movement processing can be illustrated by Haxby's distributed neural system model for face processing (Haxby et al., 2000) and O'Toole's moving face recognition neural system model (O'Toole et al., 2002). Moving faces contain static facial information (e.g., gender, ethnicity, attractiveness, and face configuration), dynamic facial information (e.g., rigid and elastic motion signals), and social information (e.g., emotion). Facial information is first processed in the primary visual processing areas, which pass different facial information into two visual processing pathways. The static facial information is processed mainly in the ventral pathway, which includes the OFA and the FFA. By contrast, dynamic facial information and social information are processed in the STS. One aspect of face processing not addressed by the prior models centers on the brain regions responsible for higher order control, including the frontal eye fields and inferior frontal gyrus. As shown in Xiao et al. (2014a), the facial movements' facilitative effect on part-based face processing involved higher brain areas, such as eye movement control. Thus, we extended the neural models from Haxby et al. (2000) and O'Toole et al. (2002) by emphasizing the role of the frontal lobe areas in processing moving faces. The processing of static and dynamic facial information would further influence higher order processing, therefore leading to the influence on eye movements and top–down face processing. Meanwhile, activity in the higher order processing areas would modulate processing in the dorsal and ventral pathways and primary visual processing areas, which optimize early face processing to adapt to task requirements (Figure 6).

## Facial movements and the development of face processing in infancy

Face processing ability develops rapidly in the first year of life (Maurer and Barrera, 1981; Le Grand et al., 2001; Nelson, 2001; Pascalis et al., 2002, 2005; de Haan et al., 2002; Kelly et al., 2005; Cassia et al., 2009; Lee et al., 2013). For example, the lateralized neural system for face processing emerges around 3–5 months of age (Guo et al., 2009; Xiao et al., 2013d). Furthermore, infants begin processing facial relational information at 4–6 months after birth (Cashon and Cohen, 2003, 2004; Schwarzer et al., 2007). Concurrently, infants gradually develop perceptual expertise for processing faces from their own ethnic background (Bar-Haim et al., 2006; Kelly et al., 2007, 2009; Liu et al., 2011; Wheeler et al., 2011). Researchers have attributed this rapid development in face processing to postnatal face experience. One common aspect of infants' face experience is that it is mostly obtained from moving face interactions in real-life situations. Given the fact that young infants tend to detect object property changes on the basis of motion signals (Bahrick and Newell, 2008), it is reasonable to assume that infants might largely rely on the dynamic aspect of facial information rather than static facial information for face processing. If this is true, the process of dynamic facial information must play a crucial role in the development of infants' face processing in the first year of life. In this section, we describe recent studies on the role of facial movement in the development of face processing in the first year of life.

### Sensitivity to facial movements

The first line of studies focus on the development of infants' sensitivity to facial movements. Studies have observed that infants are sensitive to motion signals (Kellman et al., 1986; Johnson and Aslin, 1995, 1996). In addition, they show a specific response to facial movements (Spencer et al., 2006; Ichikawa et al., 2010, 2011). The first study, to our knowledge, that revealed this specific sensitivity was conducted by Spencer et al. (2006). In this study, researchers investigated infants' ability to extract idiosyncratic facial motion characteristics from moving faces. Four- to 8-month-old infants were familiarized to a moving face avatar, which depicted joke-telling facial movements performed by one actor (Actor A told Joke 1). Following familiarization, two novel face avatars were presented side by side to examine participants' visual preference. Both face avatars presented new facial movements (Joke 2), which were not seen during familiarization. One avatar's facial movement was performed by the same actor that performed the familiarized facial movements (Actor A). The other avatar's facial movements were performed by a new actor (Actor B), whose facial movements were never shown during familiarization. This design was used to test whether infants were sensitive to an individual's specific facial movement signature. If infants were able to recognize facial motion signatures in the familiarized and test moving faces, participants should exhibit a preference for the face avatar performed by the new actor due to infants' preference for novelty (Fantz, 1964). On the contrary, if infants were not sensitive to facial movements at all, then they should attend to the two test face avatars with equal probability. The results of Spencer et al. (2006) supported the motion signature sensitivity hypothesis by revealing a significant preference for the new actor's facial movements. This finding suggests that 4- to 8-month-olds were able to encode and match an individual's facial movement characteristics. In addition, the results did not show significant age-related effects, implying that the ability to encode and represent facial movement characteristics is in place by 4 months of age and might emerge even earlier.

The existence of facial movement representation in infants has been further supported in two recent studies. Ichikawa et al. (2011) used the visual paired-comparison (i.e., VPC) paradigm to investigate infants' spontaneous preference for abstract facial movements. The abstract stimuli consisted of three black circles placed in a white head shaped contour. The three black circles were arranged to represent two eyes and a mouth. In a biologically-possible movement condition, these three black circles transformed vertically—they gradually shrunk to an ellipse, and then to a horizontal line, to emulate eye-blinking and mouth-closing movements. By contrast, a biologically-impossible stimulus exhibited a similar but horizontal circle-ellipse-line transition, which was unlikely to be perceived as a facial movement. Participants were presented with one biologically possible stimulus and one biologically impossible stimulus side-by-side. Eight-month-olds demonstrated significantly longer looking time for the biologically possible than the biologically impossible moving stimulus. This result indicates that infants around 8 months of age have already formed an abstract representation for facial movements.

In addition to Ichikawa et al. (2011), one recent study suggests that a facial movement representation might emerge around 3 months of age. Xiao et al. (2013d) compared infants' eye movement patterns for naturally and artificially moving faces. The naturally moving faces were shown counting numbers with sound removed, while the artificially moving faces were the mirror image of the naturally moving faces. The left side of the face had been flipped to the right side, and vice versa for the right side. The naturally and artificially moving faces were identical in terms of image content and facial movement magnitude. The facial movements for the naturally moving faces were ones infants are exposed to in their daily life, while the artificial ones were hardly seen in real life situations. If young infants have developed sensitivity to facial movement characteristics, they should show a face-specific eye movement pattern only on the naturally but not artificially moving faces. The face-specific eye movement pattern investigated in this study was the left visual field (LVF) bias, in which observers tend to exhibit longer looking time on the left face half (from the observer's perspective) than the right face half. This lateralized eye movement reflects the fact that face processing mostly relies on the right hemisphere, which leads to leftward eye movement (Gilbert and Bakan, 1973; Butler et al., 2005; Butler and Harvey, 2006).

Xiao et al. (2013d) found that infants around 3–5 months of age exhibited a significant LVF bias in the lower face half in the natural movement condition, but not in the artificial movement condition. In addition, for infants of 6–9 months, natural facial movements promoted the LVF bias in the whole face area. This latter result suggests that with increased moving face experience, the natural facial movements activate a stronger right hemisphere neural response, thereby leading to an even more robust LVF bias in eye movements. A recent infant neuroimaging study, using near-infrared spectroscopy technology, has provided convergent evidence by reporting a stronger neural activation in infants' right hemisphere when viewing upright moving faces than when viewing inverted moving faces (Ichikawa et al., 2010).

Taken together, these studies demonstrate that a selective sensitivity to natural facial movements emerges around 3–4 months of age. This facial movement sensitivity develops further with age, revealing the spontaneous preference for abstract facial movements, the stronger neural activation in the right hemisphere, and the capability of matching faces based on facial movement characteristics.

### Role of facial motion in the development of face recognition

The second line of research on the processing of facial movements by infants focused on the role of facial movements in the development of face recognition. One recent study suggests that facial movements facilitate face recognition as early as a few hours after birth (Bulf and Turati, 2010). This facilitation effect continues to develop throughout the first year of life (Xiao et al., 2013a). However, some studies also suggest that the introduction of movement might interfere with infants' visual attention, thus obstructing face recognition (e.g., Bahrick and Newell, 2008). We will review these recent findings regarding the effect of facial movements on the development of face processing, and discuss the underlying mechanisms.

Facial movements appear to exert mixed influence on face recognition by infants. Otsuka et al. (2009) first reported that learning a moving face could facilitate 3- to 4-month-olds' face recognition performance. Using a familiarization and VPC paradigm, Otsuka and colleagues first presented infants with either a moving face video or a static face picture for 30 s. Then, a new static face image was presented along with an image of the previously seen face, side-by-side. The results showed that moving faces led infants to accurately recognize the previous face. However, infants were unable to recognize the previous face when they were familiarized with a static face picture. These results indicate that 3- to 4-month-olds are able to utilize facial movements to enhance face recognition. The authors proposed that a moving face contains a more robust face representation than a static face picture, which accounts for the superior recognition performance.

The Otsuka et al. (2009) finding is corroborated by a study examining the role of rigid facial movements in face recognition by newborns. Bulf and Turati (2010) reported that newborns under 100 h old could recognize a face from a new viewpoint if they were familiarized with a coherent face transitioning through continuous movement from one viewpoint to another. By contrast, newborns who were familiarized with static face pictures were unable to discriminate a new face from the familiarized one. In addition, in accord with the newborn results, Otsuka et al. (2012) reported that rotating faces, but not static ones, led 3- and 4-month-olds to prefer upright to inverted Mooney faces—two-toned face images used to examine holistic face processing (Mooney, 1957; Farzin et al., 2009).

These studies converge to suggest that facial movements improve face recognition by infants, which might develop right after birth. However, in contrast to the beneficial effects of facial movement, studies have also shown that facial movement may impede infant face recognition performance when more complicated movements are introduced. For example, Bahrick et al. (2002) reported that 5-month-olds were unable to recognize faces when they were familiarized with a video in which an actor performed actions such as blowing bubbles or brushing their hair. They also found that 5-month-olds could recognize faces when familiarized with static faces. The results suggest that bodily or object motion signals might distract infant attention from processing facial information properly, thereby leading to a non-preference (Bahrick and Newell, 2008; Bahrick et al., 2013).

Additional studies have indicated that presenting a dynamic talking face results in neonates preferring that face over a novel face, whereas a static face familiarization procedure led to a novelty preference (Coulon et al., 2011; Guellaï et al., 2011). The familiarity preference induced by moving faces indicates that the representation of moving faces by infants was relatively vague and only partially formed (Hunter and Ames, 1988; Cohen, 2004). Bahrick et al. (2002) interpreted this interference effect by suggesting that motion distracts the limited attentional resources of infants from face processing, which results in poorer recognition. To summarize, in the first year of life, young observers already exhibit sensitivity to specific facial movements; however, the effect of facial movement on face recognition by infants is unclear.

The findings that face recognition by infants can be either facilitated or impeded by facial movements are in strong contrast to the relatively consistent facilitation effect observed in adult studies. The mixed results in the infant studies might be caused by differences in the type of face movement presented in the different studies, the investigated age, or individual differences in processing moving faces. With regard to differences in facial movement, some studies examined the role of rigid facial movements, such as head rotation (e.g., Bulf and Turati, 2010; Otsuka et al., 2012), whereas others focused on elastic facial movements associated with emotions such as smiling (e.g., Otsuka et al., 2009). Still other studies examined elastic facial movement associated with talking (e.g., Coulon et al., 2011; Guellaï et al., 2011), animation of abstract faces (Spencer et al., 2006), or body movements (e.g., brushing hair and brushing teeth, Bahrick et al., 2002). Given that different types of facial movements might result in different influences on face recognition, the interpretation of the set of studies taken together is problematic.

Participant age is another possible confounding factor in interpreting the effect of facial movements on face recognition performance. To our knowledge, most of the infant studies have examined the effect of facial movements before 6 months of age, and each study focused on only one specific age group, such as newborns (e.g., Bulf and Turati, 2010; Coulon et al., 2011; Guellaï et al., 2011), 3- to 4-month-olds (e.g., Otsuka et al., 2009, 2012), or 5- to 6-month-olds (e.g., Bahrick et al., 2002). Because of the differences in age groups tested, it is difficult to compare results across studies. Moreover, considering the previously mentioned stimulus inconsistencies across the studies, it is difficult to track the effect of facial movement during infant development.

Individual differences in the processing of moving faces might also contribute to the mixed effects of facial movement. The ability to process moving faces is likely still to be under development during infancy. In one recent study that used static faces, the face recognition performance of infants was closely related to their fixation transition frequency during habituation: a greater frequency of transitions led to novelty preference and a lower frequency of transitions led to familiarity preference (Gaither et al., 2012). When one considers the implications of the Gaither et al. results for studies examining the effect of movement on face recognition by infants, it is possible that for some infants, moving face parts are distracting; these infants may display fixation that will stick to a particular moving part without moving their fixation to other parts within the face, resulting in poorer face recognition (e.g., a familiarity preference). In contrast, for other infants, moving face parts may enhance encoding of the entire face by stimulating fixation shifts, thereby leading to improved face recognition (e.g., a novelty preference). Thus, different infants may have different eye movement patterns when viewing dynamically moving faces and these different patterns may be closely linked to their subsequent recognition of faces. To date, no evidence exists to support this intriguing hypothesis, because prior studies that used moving faces examined only infant visual scanning during face encoding (e.g., Hunnius and Geuze, 2004; Wheeler et al., 2011; Lewkowicz and Hansen-Tift, 2012) or only tested their face recognition performance (e.g., Otsuka et al., 2009, 2012; Bulf and Turati, 2010).

To summarize, due to the inconsistency in the moving face stimuli, participant age, and possible individual difference in processing moving faces, previous infant studies were unable to provide a comprehensive account for the effect of facial movements in the development of face processing in the first year of life.

To address these inconsistencies in the previous literature, one recent study using silent chewing and blinking moving faces as stimuli investigated the development of moving face processing in infancy. Xiao et al. (2013a) used the familiarization and VPC paradigm to examine the role of facial movements in face recognition at the ages of 3, 6, and 9 months. Similar to the procedure applied in Otsuka et al. (2009), infants were first familiarized with a moving (chewing and blinking) or a static face, which was followed with a pair of static face pictures: one previously shown and one new face. Infants' eye movements were recorded during the familiarization and test phases. Two eye movement events were analyzed: the accumulative proportional looking time and the inter-feature (i.e., eyes, nose, mouth) fixation shifts.

**Figure 7:**
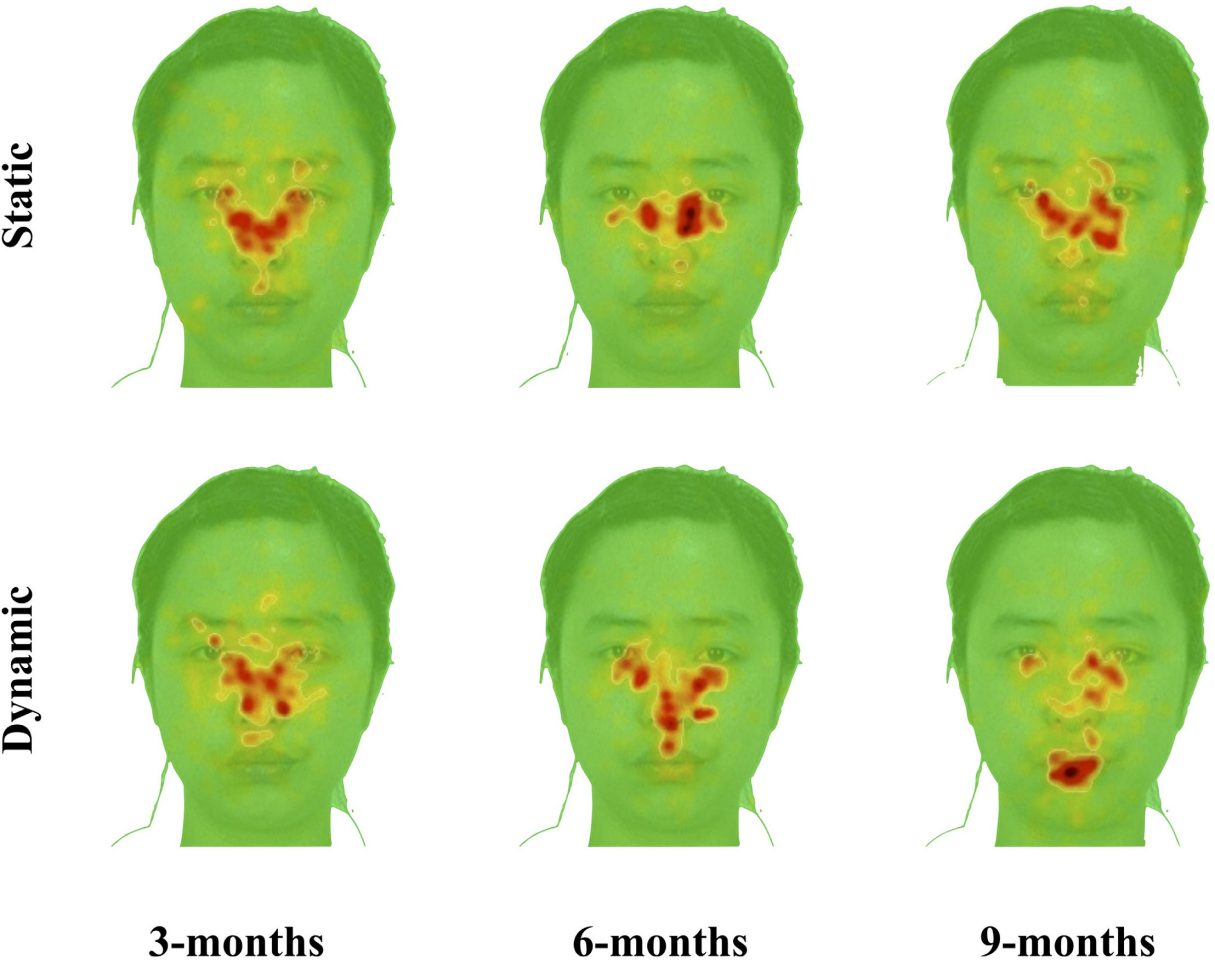
**Spatial distribution of fixations during familiarization as revealed through the iMap procedure**. The areas in warm colors (i.e., yellow and red) depict the regions being fixated on. Within these fixated areas, the redder the color is, the longer the time this area is fixated on. The plots show that the 3-month-olds fixated mainly on the center of the face and this fixation pattern is similar on the moving and static faces; by contrast, 6- and 9-month-olds' fixations are more expansive on the moving faces than on the static faces.

**Figure 8:**
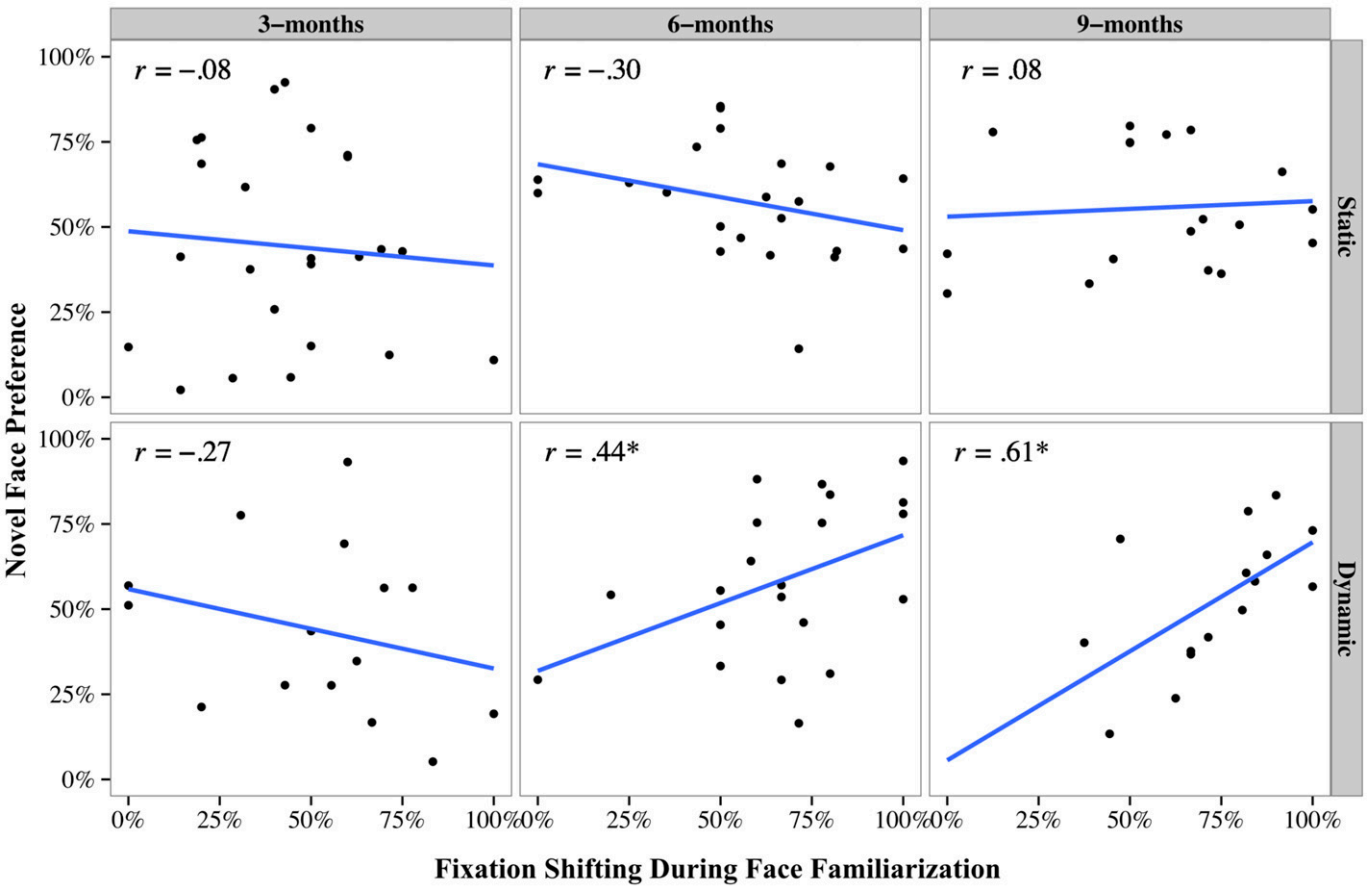
**Correlation between fixation shifting (x-axis) and novelty preference (y-axis) for the static and dynamic conditions in the 3 age groups**.

Xiao et al. (2013a) reported significant differences in eye movement patterns when infants looked at moving and static faces. With increased age, infants looked longer at the mouth region of the moving than static faces, and less at the eye region of moving than static faces. Moving faces also activated more fixation shifts between facial features than static faces. In contrast, infants fixated mostly at the center of static faces, regardless their age (Figure 7). Most importantly, a significant positive correlation between the frequency of fixation shifts and infants' face recognition from 6 months onward was revealed. Infants who shifted fixations more frequently during the moving face familiarization showed better recognition. By contrast, infants who shifted fixations less frequently failed to recognize the previously seen face and differentiate it from the new face (Figure 8). This correlation was only observed in the moving face condition, but not in the static face condition, suggesting a role for facial movements in modulating infant face recognition performance. These results indicate that infants exhibit a distinctive eye movement pattern in processing moving faces, which is linked to their face recognition performance. For some infants, facial movements activate fixation shifting across the face thereby facilitating face recognition, whereas for other infants, facial movements engender fixations to certain face regions and inhibit fixation shifting, which interferes with face recognition.

To conclude, facial movements play an important role in the development of face processing in infancy. Through moving face experiences in real-life situations, infants form specific representations for facial movements around 3–4 months of age. Facial movements further affect infants' face recognition performance, in either a facilitative or a detrimental way. Although facial movement influences the development of face processing in infancy, it has yet to reach the level of flexibility and efficiency demonstrated in adult processing of moving faces. It may be that the effect of facial movement develops continuously through infancy to adulthood.

## Future directions

Despite evidence of a significant role for facial motion in face processing and its development, it is still premature to conclude that we process moving faces in a fundamentally different way than static faces. Here, we propose several directions for future studies to further elucidate the mechanisms underlying moving face processing.

On the basis of the representation of facial movements, it becomes easier to recognize familiar moving faces. However, little is known regarding the mechanism through which we form and store a representation for facial movement information. The supplementary information hypothesis assumes that dynamic facial representation forms as faces become familiar to observers (O'Toole et al., 2002). Prior studies that examined the dynamic face representation focused on faces that participants already were familiar with; however, to our knowledge, no study has examined the process through which the representation of facial movement is formed. To understand the factors contributing to this process would be an important focus for future studies. A developmental approach may be considered to examine the formation of representations for moving faces. Moreover, we can examine the formation of representations for facial movement using a laboratory training approach to examine the relevant factors in controlled contexts.

In a recent revision of the representation enhancement hypothesis, O'Toole and Roark (2010) posited that the representation enhanced by facial movement might not be limited to the static components of face representation. Facial movements may also enhance a general dynamic face representation, which could only be accessed with facial movements. This is demonstrated in a task that displays facial motion information in both learning and test faces. Roark et al. (2006), for example, presented observers with videos of whole-body movement of unfamiliar individuals, and then tested their face recognition with a moving face or a static face image. Results showed that observers recognized moving faces better than static ones, even though the moving faces depicted different facial movements from those in the learned faces. The findings indicate that the congruent learning and testing pairs (i.e., motion–motion) led to better recognition than the incongruent pairs (i.e., static–motion or motion–static). Relatedly, Lander and Davies (2007) reported a similar result that observers could accurately recognize faces in motion if these faces were first learned in motion. An important difference between this general motion representation and the idiosyncratic facial movement representation (the supplementary hypothesis) is that it does not require a specific movement pattern to be activated. As long as a face is moving, this general motion representation would be activated and lead to advanced recognition performance. However, more studies should be conducted to further examine the arguments for a general representation of moving faces as one recent study failed to show enhanced recognition for moving faces over static faces when faces were learned in motion (Butcher et al., 2011).

As for the role of facial movements in face processing optimization, current evidence is derived from relatively limited paradigms, such as the composite effect and the Garner classification task. Future studies should include additional paradigms to examine the universality of the facilitative effects of facial motion. For example, due to the limitation of the composite paradigm, we have only been able to examine the effect of facial movements in recognizing static faces. In real life contexts, however, both face learning and recognition occur in interactions with moving faces. Studies using moving faces as test faces would allow us to better understand the influences of facial movements in learning and recognizing faces. The part-whole task (Tanaka and Farah, 1993; Tanaka et al., 1998), another method to gauge holistic face processing, would be an ideal paradigm to supplement the composite effect in testing moving face recognition. In addition, applying eye tracking technology and analyses of individual differences could further elucidate the mechanisms that underlie the effect of facial movements.

From a developmental perspective, facial movements have been found to contribute to the development of face processing in the first year of life. Studies have reported that the eye movement patterns in processing moving faces coincide with the development of other aspects of face processing development in infants, such as the processing of facial configuration information (Bhatt et al., 2005; Scott, 2006). Future studies may consider investigating whether facial movements contribute to the emergence of other aspects of face processing to understand the nature of face processing development in real-life contexts. Moreover, results from infant studies suggest that the effect of facial movements may underlie further development from infancy to adulthood. To our knowledge, very few moving face studies have focused on the childhood period. Additional studies should focus on this period to complete the developmental trajectory of moving face processing.

This article mainly focused on the behavioral outcomes of moving face processing. We should not ignore the neural activities that underpin the behavioral findings. To our knowledge, few neuroimaging studies have been conducted to understand the distinctive neural activities in processing moving vs. static faces (Fox et al., 2009; Schultz and Pilz, 2009; Ichikawa et al., 2010; Pitcher et al., 2011; Schultz et al., 2013). These studies, however, have consistently revealed increased activation in the STS region in the right hemisphere that is driven by the processing of moving faces. However, it is still unclear what the specific role of the right STS is in processing facial movement information, and what the potential links between the neural activities and facial motion related performance improvements are. Future neuroscience investigations might be considered to further probe the distinctive neural activities associated with moving face processing.

## Summary and conclusions

We reviewed recent behavioral findings regarding moving face processing. These studies demonstrate that the processing of moving faces is distinctive from what we have learned from static face processing studies. The distinctive aspects of moving face processing were first revealed in their effects on face representation, which has been summarized in two hypotheses: the supplementary information hypothesis and the representation enhancement hypothesis. In addition, facial movements have been recently found to optimize face processing strategies, reflecting increased flexibility to respond to changing task requirements, and thereby facilitating performance. Lastly, facial movements play a significant role in shaping the development of face processing in the first year of life. Infants develop representations for facial movement characteristics, which enable more efficient face recognition. In summary, through comparison of moving and static face processing, it appears that studies of moving face processing take us beyond what we have learned from investigation of static faces. In future studies, we must not ignore the dynamic aspect of facial information, as it might provide crucial insights into the nature of face processing and its development in real life contexts.

### Conflict of interest statement

The authors declare that the research was conducted in the absence of any commercial or financial relationships that could be construed as a potential conflict of interest.
